# Blackburn and a Clean Milk Supply

**Published:** 1924-03

**Authors:** 


### BLACKBURN'S CLEAN MILK.

The health authorities of Blackburn, says the *Preston Guardian*, are doing an excellent work in encouraging the production and consumption of clean milk. A CleanMilk Competition was inaugurated in 1922, when Alderman J. Fielding, chairman of the Health Committee and Mayor of the borough for that year, offered a silver challenge cup for the competitor scoring the highest number of points, the holder also receiving a permanent memento in the form of a gold medal.

### The Food Value of Milk.

Dr. W. A. Daley, Blackburn's medical officer of health, while fostering the efforts to raise the quality of the town's milk supply to a high standard, is also helpful to producers in endeavouring to increase demand, and as the result of his communications with the Milk Publicity Council, an experiment to prove the food value of milk is now being carried out with school children in a poor district of the town. Forty children, most of whose parents are in receipt of poor-law relief, have been selected, this choice being made because of the financial conditions of the home, and presumably the quantity and nutritive values of the foods they receive will be standardised as nearly as possible. For the purposes of the test, the children have been divided into two groups of 20 each. Group I. receive a pint per day of Grade A milk, half in the morning and the other half in the afternoon, with two biscuits in addition. The milk is drawn through straws straight from the bottle. Group II. get the biscuits without milk, but at the end of three months, the order will be changed, and they will get the milk diet which will be withheld from Group I. The children are weighed carefully every week, and the results so far are distinctly encouraging, Blood tests already made show some of the children to be anæmic, and before the experiment concludes these tests will be repeated to ascertain whether there is any improvement in quality."

